# Sural flap reconstruction of lateral malleolus following undifferentiated pleomorphic sarcoma resection with 1-year follow-up

**DOI:** 10.1093/jscr/rjae447

**Published:** 2024-07-09

**Authors:** Motaz Saifi, Omar Younis, Ibrahim R Nour, Saad Abuzahra, Mamoun Mansour, Mohammed Hasan

**Affiliations:** Department of Medicine, An-Najah National University, PO Box 7, Nablus, West Bank, Palestine; Department of Medicine, An-Najah National University, PO Box 7, Nablus, West Bank, Palestine; Department of Orthopedic Surgery, Al-Makassed Charitable Hospital, Mount of Olives in East Jerusalem, West Bank, Palestine; Department of Medicine, An-Najah National University, PO Box 7, Nablus, West Bank, Palestine; Department of Orthopedic Surgery, Al-Makassed Charitable Hospital, Mount of Olives in East Jerusalem, West Bank, Palestine; Department of Medicine, An-Najah National University, PO Box 7, Nablus, West Bank, Palestine; Plastic and Reconstructive Surgery, An-Najah National University Hospital, Asira Street, Nablus, West Bank, Palestine

**Keywords:** undifferentiated pleomorphic sarcoma, malignant fibrous histiocytoma, soft tissue sarcoma, reverse sural artery flap, reconstruction

## Abstract

We report a case of a 54-year-old female who presents with a gradually expanding mass at the right lateral malleolus. The diagnosis of undifferentiated pleomorphic sarcoma was made after a histopathological examination of the mass following a wide tumor excision. The defected soft tissue area was reconstructed using a local flap, reverse sural artery flap. Following the surgical management, multiple radiotherapy sessions were completed. The patient’s follow-up result showed no signs of local recurrence or metastasis, and the wound was well-healed with no complications other than paresthesia in a small area at the posterolateral aspect of the ankle under the lateral malleolus. This case represents a rare form of malignant neoplasm and emphasizes the effectiveness and safety of the reverse sural artery flap reconstruction technique, especially in places where microsurgery is unavailable or when the patient’s status does not allow for prolonged anesthesia.

## Introduction

Undifferentiated pleomorphic osteosarcoma (UPS), previously called malignant fibrous histiocytoma, is a rare, mesenchymal, high-grade type of soft tissue sarcoma that frequently affects the lower extremities [1]. UPS has non-specific clinical features; therefore, the diagnosis will be done through exclusion similar neoplasms histopathologically [2]. UPS management is similar to other high-grade extremity soft tissue sarcomas, in which wide tumor resection is the standard treatment and frequently complemented by chemotherapy and radiotherapy [3]. Nonetheless, wide resection can cause a large-scale soft tissue defect that necessitates reconstruction; hence, pedicle or free-flap reconstruction can be used for adequate tissue coverage [4]. Regardless of its popularity since the emergence of perforated-based flaps and microsurgery, reverse sural artery flap is still thought to be a safe and effective way to cover up soft tissue defects in the lower third of the limbs [5].

Herein, we report a case of right lateral malleolar undifferentiated pleomorphic sarcoma that was reconstructed using a reverse sural artery flap following tumor resection, with 1-year follow-up results.

## Case presentation

A 54-year-old female noticed a mass on the outer surface of her right ankle while wearing her clothes. However, she didn’t give it much attention until 1 month, after noticing how the mass had enlarged. The mass was gradually enlarging and was neither associated with local symptoms like pain, tenderness, or hotness nor systemic symptoms like fatigue, fever, or weight loss. The patient has had metformin-controlled type 2 diabetes for 10 years and no other medical or surgical history.

**Figure 1:**
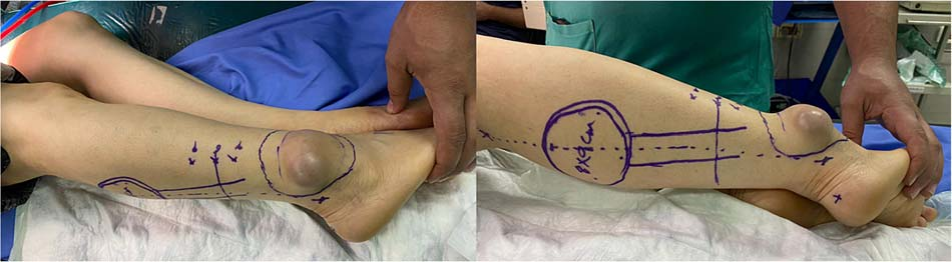
Gross views of the mass and the sural flap drawings at the operation table.

A physical examination showed a 4.5 × 7.5 cm oval mass was located at the right lateral malleolus (Fig. 1), which was firm, ill-defined, not adherent to the skin or the tissue underneath, not associated with any tenderness or hotness, negative trans-illumination test, and there were no palpable inguinal lymph nodes.

**Figure 2:**
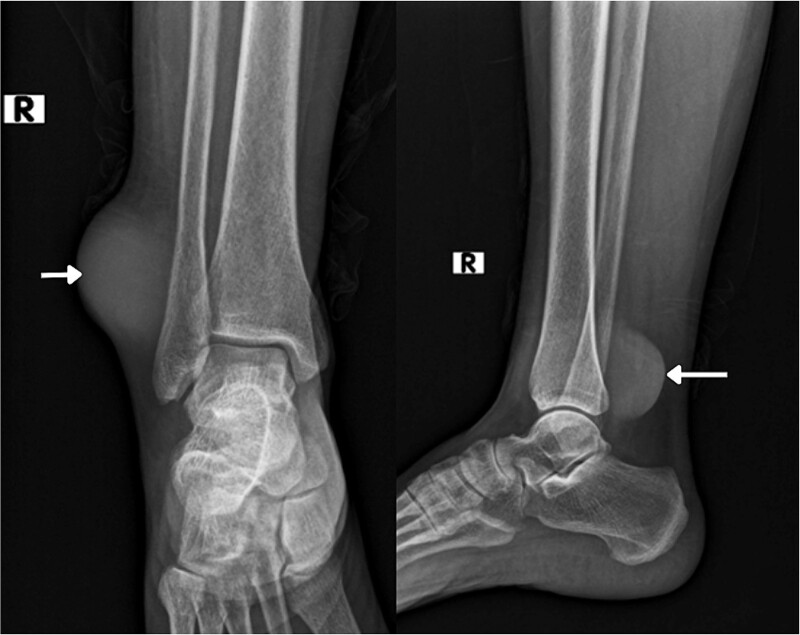
Radiological imaging pre-op.

On imaging, an ankle X-ray showed an oval soft tissue mass subcutaneously at the level of the distal fibula (Fig. 2). The MRI showed about 5.7 × 3 × 5 cm of well-defined soft tissue mass within the distal lateral compartment of the lower leg, which seems locally invasive, abutting the fibular cortex, but without evidence of fibular cortical invasion (Supplementary Fig. 1). Moreover, a whole-body CT confirmed the absence of metastasis**.** CRP, ESR, LFT, and KFT were all within the normal range, and her bone profile was normal for her age. However, hemoglobin levels were slightly decreased (10.5 g/dl). An incisional biopsy showed features consistent with UPS. Therefore, the decision was to proceed with wide tumor resection and radiotherapy sessions.

Dissection from all sides followed a circular incision over the lateral ankle with a 1 cm free margin. After discovering that the tumor was invading the fibula bone’s periosteum, all surrounding tissue was released except for it. The anterolateral cortex of the fibula was removed, leaving the bone in its original position untouched, and the excised tumor was sent to pathology. Plastic surgery hindered tissue reconstruction after tumor removal (Supplementary Fig. 2).

**Figure 3:**
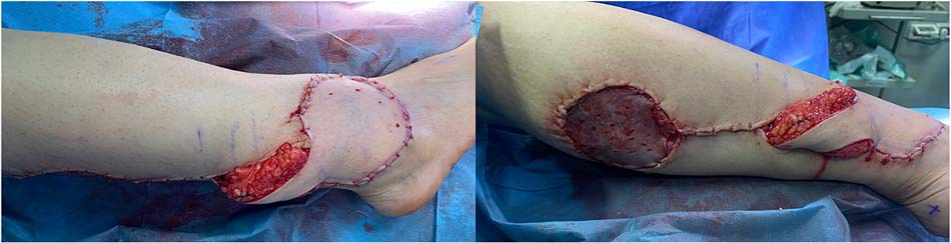
Gross views of the reconstruction area postoperatively.

In this case, the flap measured 8 × 9 cm (Fig. 1). Skin incision at the flap’s superior edge and identification of the lesser saphenous vein and sural nerve led to flap harvesting at the superolateral edge in the subfascial plane. A small skin tail was preserved when the pedicle was dissected in the subdermal plane to improve venous return. The donor site was closed with a split-thickness thigh skin graft after the flap was rotated and sutured to the defect (Fig. 3). Flap separation occurred a month later.

**Figure 4:**
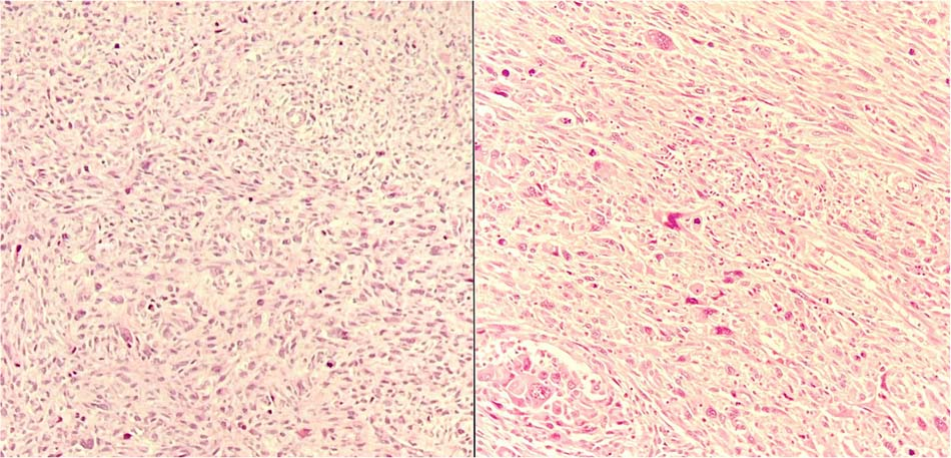
Histologic sections of the resected tumor.

The pathology examination showed that the resected tumor is a Stage 2 undifferentiated pleomorphic sarcoma according to the American Joint Committee on Cancer staging system for soft tissue sarcoma (Fig. 4) [6]. Accordingly, the patient had 28 radiotherapy sessions 4 months after the excision.

**Figure 5:**
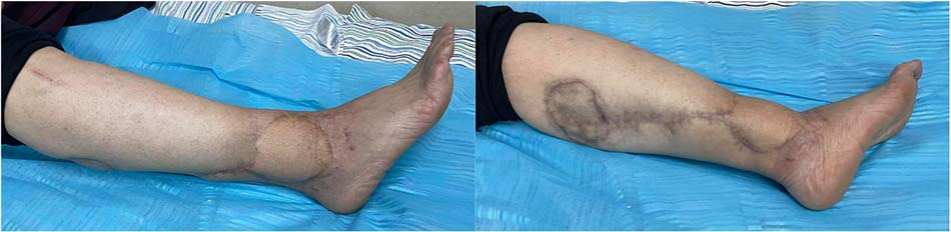
Gross views of the reconstructed area after one year of follow-up.

**Figure 6:**
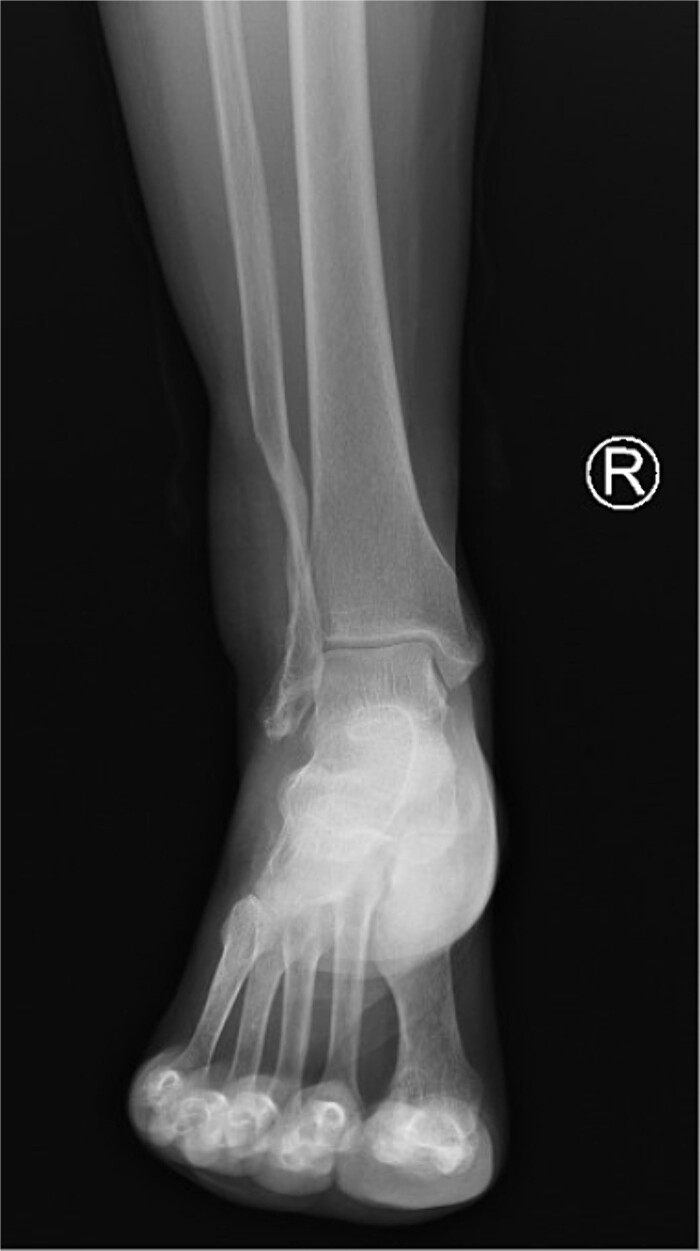
Ankle AP X-ray after 1 year of follow-up.

The patient started having regular follow-up visits 1 week after the procedure, in which at the last follow-up she had a well-healed wound, showed no scar abnormalities, and had a mildly elevated thickness compared to the surroundings (Fig. 5). The patient had paresthesia in a small area at the posterolateral aspect of the ankle under the lateral malleolus. However, there were no limitations on movement, and the patient was able to bear weight and comfortably wear shoes. Moreover, a follow-up X-ray showed no abnormalities (Fig. 6).

## Discussion

UPS is an aggressive, high-grade soft tissue sarcoma that is believed to originate from mesenchymal stem cells [1]. According to a study on 26,000 soft tissue sarcoma cases, UPS accounted for 17.1% and was the second most prevalent type after leiomyosarcoma. Moreover, the incidence was predominantly higher in white males [7]. Furthermore, UPS is most commonly seen in people aged between the fifth and seventh decade of life and is most commonly seen in lower extremities [7].

UPS has no specific clinical features that set it apart from other types of soft tissue sarcoma. These types also have a wide range of histological presentations, which makes diagnosis difficult. Therefore, diagnosis of exclusion is made during the histopathological examination [2].

It is important to note that early detection and management of UPS are critical for improvement in the overall prognosis. A specific report regarding UPS found that the local recurrence rate was 15% and was significantly associated with inadequate surgical margins and advanced age; furthermore, metastasis rate was 37.6% and was substantially relevant with tumors sized 5 cm or more. However, invasion depth and advanced staging can also contribute to these numbers [8].

Wide tumor excision is considered to be the main approach in the management of UPS and other high-grade soft tissue sarcomas. Nonetheless, wide resection can pose a challenge of inadequate soft tissue covering that cannot be managed by simple skin grafting or primary wound closure, necessitating reconstruction with sufficient skin coverage, which can be done by pedicle or free-flap [4].

The reverse sural artery flap is one of the reconstruction techniques used for leg defects at the distal third using a local flap. The sural flap is considered a safe technique [5]. It also has a shorter recovery time, a large range of rotation, and provides the best thickness without losing major artery arteries for reconstructing the lower third of leg defects [5, 9, 10].

In our case, during the tumor resection process, we found that the tumor was invading only the periosteum of the fibula; hence, we found it unnecessary to remove the whole distal third of the fibula. Instead, we resected longitudinally the outer half of the distal fibula third, leaving the fibular body in its original position to preserve the original ankle shape and stability. Therefore, we believe that the sural flap remains a safe and effective option for reconstructing lower limb defects, particularly in situations where patient status does not fit for prolonged surgical procedures or in an understructured healthcare system.

## Supplementary Material

Supp_Figures_Legend_20_3_2024_rjae447
