# Supplementary material for: Sural flap reconstruction of lateral malleolus following undifferentiated pleomorphic sarcoma resection with 1-year follow-up
Source: J Surg Case Rep. 2024 Jul 9;2024(7):rjae447. doi: 10.1093/jscr/rjae447 (PMC11232055; doi:10.1093/jscr/rjae447)
Supplement: Supp_Figures_Legend_20_3_2024_rjae447 [file supp_figures_legend_20_3_2024_rjae447.docx]

**
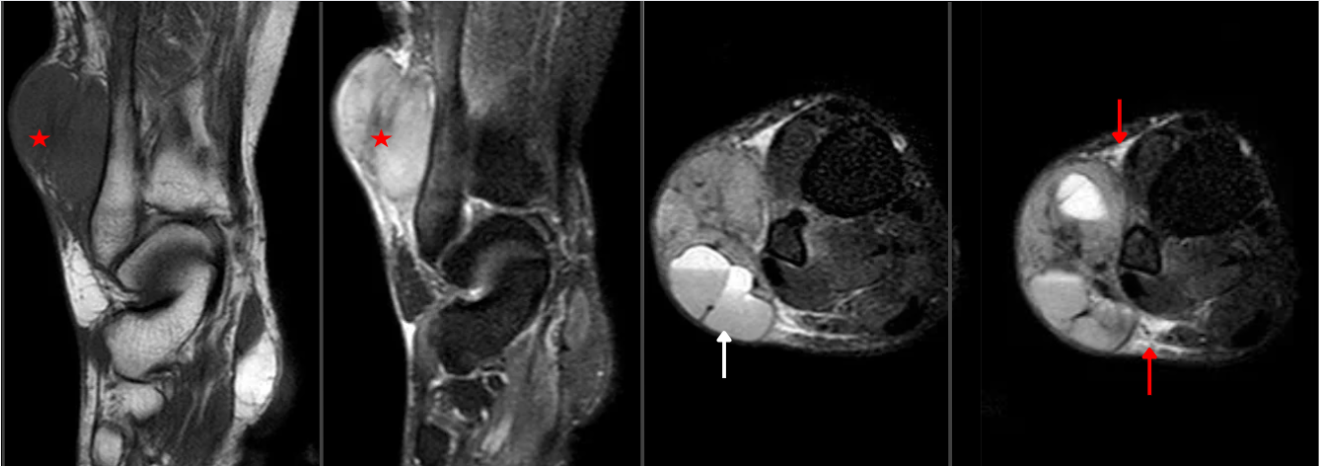
**

**Supp. Figure 1:**

MRI Coronal T1 WI and PD fat sat, respectively, show a well-defined soft tissue mass within the distal lateral compartment (red stars) of the lower leg abutting the distal fibular cortex with low signal intensity on T1 WI and heterogeneous intermediate to high signal intensity on the PD fat sat sequence. MRI axial PD fat sat images at two different levels show areas of fluid levels within the lesion (vertical white arrow) and peritumoral hyperintensity (vertical red arrows).

* PD: Proton Density.

**
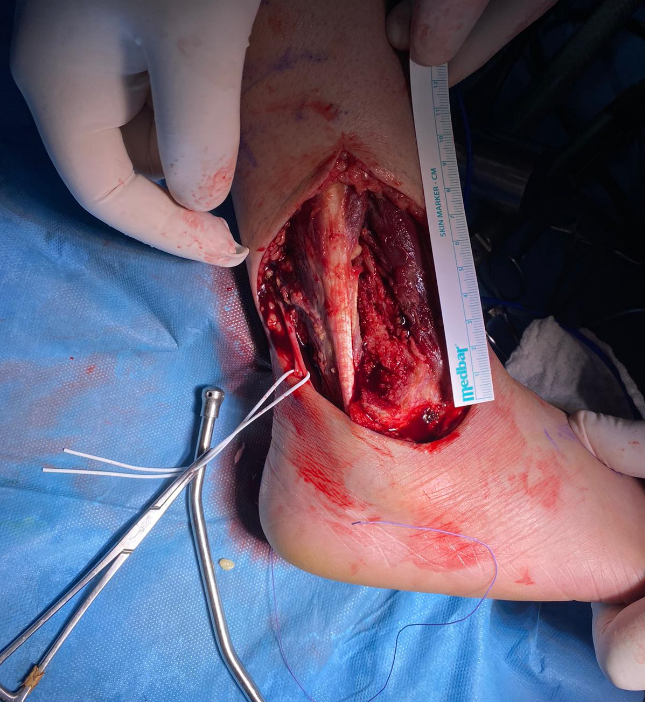
**

**Supp. Figure 2:**

Complex defect of the lateral malleolar area after the tumor excision.
